# Meaning in life and influencing factors among Chinese nurses: a multi-center cross-sectional study

**DOI:** 10.3389/fpsyg.2025.1662466

**Published:** 2026-01-27

**Authors:** Hongli Ma, Siqi Wu, Rui Li, Lei Luo, Huirong Yuan, Youwen Gong, Hongling Zheng

**Affiliations:** 1 Breast Cancer Ward 1, Sichuan Clinical Research Center for Cancer, Sichuan Cancer Hospital & Institute, Sichuan Cancer Center, University of Electronic Science and Technology of China, Chengdu, China; 2 Department of Neurosurgery, Changde Hospital, Xiangya School of Medicine, Central South University (The First People's Hospital of Changde City), Changde, China; 3 Department of Nursing, Sichuan Clinical Research Center for Cancer, Sichuan Cancer Hospital & Institute, Sichuan Cancer Center, University of Electronic Science and Technology of China, Chengdu, China; 4 General Affairs Department, Sichuan Clinical Research Center for Cancer, Sichuan Cancer Hospital & Institute, Sichuan Cancer Center, University of Electronic Science and Technology of China, Chengdu, China

**Keywords:** anxiety, attitude to death, cross-sectional studies, nurses, value of life

## Abstract

**Background:**

Meaning in life is a comprehensive understanding of the significance and aim of one's own existence. There is limited research that specifically focuses on meaning in life for nurses, whose work may expose them to substantial workloads, intricate interpersonal dynamics, frequent exposure to death, round-the-clock shifts, and workplace violence. Meaning in life can be beneficial for nurses' psychological well-being and indirectly enhance patients' quality of care. This study explored the impact of sociodemographic factors, death anxiety and attitudes toward death on the meaning in life for Chinese nurses in Sichuan Province.

**Method:**

Between February to April 2024, a multicenter cross-sectional survey involving 1698 Chinese nurses from 60 hospitals across Sichuan Province was conducted. A self-designed sociodemographic survey, Meaning in Life Questionnaire, Templer-Death Anxiety Scale, and Frommelt Attitudes Toward Care of the Dying Scale Form B were used to collect data. A multiple linear regression model was used to identify significant variables related to meaning in life based on sociodemographic factors, death anxiety, and attitudes toward death.

**Result:**

A total of 1,388 valid questionnaires were obtained. The average score for meaning in life among Chinese nurses was 50.37 ± 9.30. Having religious beliefs, attitudes toward death, married status, senior title, secondary grade B and below hospital, and being a head nurse were statistically significant for meaning in life, and these factors explained 24.9% of the variance (*F* = 25.364, *P* < 0.001).

**Conclusion:**

The study revealed that nurses in Sichuan Province have a moderate level of meaning in life, which is influenced by their attitudes toward caring for dying, marital status, religious beliefs, title, hospital grade, and position. Administrators and regulatory bodies could leverage these personality traits to develop targeted interventions.

## 1 Introduction

The term “meaning in life” describes individual comprehensive understanding of the significance and aim of one's own existence, and the pursuit of meaning in life is the fundamental driving force behind human behavior (Frankl, 1966; Yu et al., 2022). Steger proposed that a sense of meaning in life encompasses an individual's recognition and appreciation of the significance of life. This includes two dimensions, a sense of being meaningful and a sense of seeking meaningfulness, which represent cognitive and motivational aspects (Steger et al., 2006b). The term “meaning in life” describes an individual's comprehensive understanding of the significance and purpose of their existence (Czekierda et al., 2017), as well as the motivation to pursue that significance (Mulahalilović et al., 2021). Based on previous literature and the context of Chinese clinical nursing, in this study, we define meaning in life as the degree to which nurses perceive their lives as purposeful, valuable, and worth living, encompassing both the recognition of life's significance (cognitive aspect) and the active pursuit of meaningful experiences (motivational aspect) (Li et al., 2019). This definition provides a practical framework for examining how sociodemographic factors, death anxiety, and attitudes toward death influence nurses' perception of life meaning in clinical settings (Chen et al., 2022; Maffoni et al., 2021; Mutuyimana and Maercker, 2024; Wang et al., 2021).

Extensive research has been conducted to investigate the influence of meaning in life on individuals from a sociological perspective, however, few studies have examined the meaning in life from a medical perspective. Medical research on meaning in life has primarily focused on exploring its significance for patients and medical students (Kerry et al., 2023; Martinez-Calderon et al., 2023, 2024; Maynard et al., 2024; Sun et al., 2024). There is limited research that specifically focuses on nurses confronted with substantial workloads, intricate interpersonal dynamics, frequent exposure to death, round-the-clock shifts, and workplace violence (Cho et al., 2022; Christensen et al., 2024). These negative emotions often lead nurses to question the meaning and value of both their lives and work. Meaning in life can be beneficial for nurses' psychological wellbeing and indirectly enhance the quality of care provided to patients. Further research is necessary to deepen our understanding and explore more effective intervention methods.

Death can be viewed as the ultimate evidence of the loss of meaning in life. Contemplating mortality can instigate introspection and cause individuals to question the extent of their own purpose and meaning in life (King and Hicks, 2021). Because traditional Chinese culture views death as a taboo, death anxiety tends to occur during the process of contemplating death. Extensive research has been conducted to investigate the connection between death anxiety and meaning in life. Most of these studies focused on patients, revealing a negative correlation between death anxiety and the perception of meaning in life (Dursun et al., 2022; Li et al., 2024; Maffly-Kipp et al., 2024; Yan et al., 2024). Similar studies have been conducted with nursing students, with findings consistent with those of previous patient studies (Xu and Yu, 2024; Yuan et al., 2024). However, it is worth noting that one cross-sectional study conducted in India demonstrated that higher levels of meaning in life among nursing faculty and students were associated with increased severity of death anxiety (Latha, 2013). Consequently, further validation of the relationship between death anxiety and meaning in life is necessary, as only a limited number of studies have explored this in the context of nurses.

Attitude toward death is another crucial factor closely intertwined with the perception of meaning in life. Meaning in life has been found to have a detrimental effect on attitudes toward palliative care among undergraduate nursing students (Xu and Yu, 2024), as well as a negative impact on attitudes toward hospice care among nursing students (Yu et al., 2022). However, there is a dearth of additional research investigating the connection between meaning in life and attitudes toward death, specifically within the nursing population. These findings highlight the need for further research to elucidate this relationship.

According to meaning-in-life theory, an individual's sense of meaning arises from their interpretation of life experiences, personal values, and existential reflection (Steger et al., 2006b). This theoretical framework highlights that meaning in life is influenced by both internal psychological factors—such as beliefs, attitudes, and emotional reactions—and external contextual conditions. In the nursing profession, frequent exposure to suffering, critical illness, and patient death may heighten existential awareness and trigger death-related cognitions, making death anxiety and attitudes toward caring for dying patients theoretically relevant predictors of meaning in life (Chen and Gao, 2021).

Understanding the determinants of meaning in life among Chinese nurses has both theoretical and practical significance. Theoretically, it contributes to occupational health psychology by clarifying how personal, professional, and cultural factors interact to shape life meaning in a high-stress clinical environment (Aslan et al., 2022). Practically, identifying modifiable factors that enhance life meaning can inform interventions—such as death education, psychological support, and organizational strategies—to improve nurses' wellbeing, job satisfaction, and quality of patient care (Mian and Rejnö, 2024). Attitudes toward death and death anxiety are particularly relevant in this context. Exposure to patient mortality can trigger existential reflection and emotional distress, influencing nurses' perceived purpose and value in their work. Previous studies suggest a negative correlation between death anxiety and meaning in life among patients and nursing students (Dursun et al., 2022). Positive attitudes toward caring for the dying may serve as a protective factor, enabling nurses to engage more meaningfully with end-of-life care and maintain a stable sense of life meaning. In the Chinese cultural context, where death is traditionally a taboo topic, nurses may experience additional internal conflict when confronting mortality, amplifying death anxiety and potentially diminishing life meaning (Yang and Wu, 2021). Understanding these culturally specific mechanisms is essential for developing tailored interventions that address both psychological and professional challenges in nursing practice.

Taken together, these considerations highlight a clear research gap: few studies have systematically examined meaning in life among practicing Chinese nurses, especially with attention to death-related factors within the local cultural context. This study aims to address this gap by investigating the current level of meaning in life and its influencing factors, including sociodemographic characteristics, death anxiety, and attitudes toward death, among nurses across multiple hospitals in Sichuan Province.

## 2 Methods

### 2.1 Design and ethical review

This is a multi-center cross-sectional study. This study was approved by the Ethics Committee of Sichuan Cancer Hospital (approval number: SCCHEC-02-2023-035).

### 2.2 Participants

Data for this study were collected using convenience sampling from 60 hospitals across Sichuan Province between February to April 2024. A total of 1,698 nurses completed the survey, with 1,388 valid questionnaires were included in the final analysis, yielding an effective response rate of 81.7%. The sample size was determined based on the principle of multivariate regression analysis, which requires at least 10–15 participants per independent variable. Considering that our study included 20 potential predictors in the regression model, a minimum of 200–300 participants would be required. Our final sample of 1,388 participants therefore exceeds this minimum, providing adequate statistical power to detect meaningful associations. The inclusion criteria were as follows: (1) nurse practitioners and (2) nurses engaged in nursing work. The exclusion criteria were as follows: (1) nurses who were on any form of official leave during the survey period, including sick leave, maternity leave, personal leave, or vacation leave; (2) nurses with a history of diagnosed mental illness or currently undergoing related treatment; and (3) nurses who chose not to participate in this study.

### 2.3 Tools

#### 2.3.1 Socio-demographic questionnaire

The questionnaire was developed on the basis of a literature search and extensive discussions within the research team. It encompassed 20 items, including gender, age, ethnic group, marital status, educational level, title, department, grade of hospital, responsible institution, nature of the hospital, location of hospital, position, average monthly income, whether a specialized nurse, religious belief, bereavement within 1 year, average number of terminally ill patients cared for per month, whether death education training had been received, Frommelt Attitudes Toward Care of the Dying Scale Form B (FATCOD-B), and Templer-Death Anxiety Scale (T-DAS).

In China, hospitals are classified according to the national three-tier hospital grading system issued by the National Health Commission (Shi et al., 2021). Tertiary Grade A (Class III-A) is given to the highest-level hospitals that provide comprehensive and specialized medical services, engage in teaching and research, and manage complex or critical conditions. Tertiary Grade B (Class III-B) is large regional hospitals offering comprehensive services but with slightly fewer resources or research capacity than Grade A institutions. Secondary Grade A (Class II-A) is mid-size hospitals providing regional medical services and responsible for the diagnosis and treatment of common and frequently occurring diseases. Secondary Grade B and below (Class II-B or Class I hospitals): are hospitals with more limited resources that primarily offer basic medical and public health services to local communities. These definitions follow the national hospital classification standards and were used to categorize the institutions included in this study.

#### 2.3.2 Meaning in life questionnaire (MLQ)

The MLQ was initially developed by Steger et al. (2006a), translated into Chinese, and validated by Wang (2013). The Chinese version of this scale includes ten items divided into two subscales: (1) the presence of meaning (MLQ-P), consisting of five items, and (2) the search for meaning (MLQ-S), consisting of five items. Participants rated each item on a 7-point Likert scale, with a total score ranging from 10 to 70. The MLQ demonstrated good internal consistency with a Cronbach's coefficient of 0.830 for the total scale. Additionally, it showed acceptable test-retest reliability with a coefficient of 0.639. In the present study, the total MLQ scale showed a Cronbach's α of 0.87, with α values of 0.84 for the presence of meaning subscale and 0.82 for the search for meaning subscale.

#### 2.3.3 Frommelt attitudes toward care of the dying scale form B (FATCOD-B)

The FATCOD-B was initially developed by (Frommelt, 1991, 2003) and later translated and revised into Chinese by Liping (2016). The Chinese version of the FATCOD-B consists of 29 items and 6 subscales. It is scored on a Likert 5-point scale, where 14 positive items are scored from strongly agree to strongly disagree (5–1 points), and 15 negative items are scored in the opposite direction, from strongly agree to strongly disagree (1–5 points). The total score ranges from 29 to 145, with higher scores indicating a more positive attitude toward end-of-life care and a greater likelihood of engaging in proactive caregiving behaviors for dying patients. The scale demonstrated good internal consistency with a Cronbach's alpha coefficient of 0.79 and high content validity with a score of 0.92 (Liping, 2016). In the current sample, the total FATCOD-B scale demonstrated a Cronbach's α of 0.90, and subscale α coefficients ranged from 0.72 to 0.88, indicating good reliability.

#### 2.3.4 Templer-death anxiety scale (T-DAS)

The T-DAS is a measurement tool that was developed by Professor Templer at California University in 1967 (Templer, 1970). It was translated into Chinese, validated by Yang (2011), and consists of 15 items. Participants rate each item on a 5-point Likert scale, with nine positively scored items ranging from 1 (strongly disagree) to 5 (strongly agree) and six negatively scored items ranging from 5 (strongly disagree) to 1 (strongly agree). The total score on this scale ranges from 15 to 75, with scores above 35 indicating high levels of death anxiety. The Chinese version of the scale demonstrated good internal consistency with a Cronbach's alpha coefficient of 0.71 and a test-retest reliability coefficient of 0.83. In the present study, the total T-DAS scale produced a Cronbach's α of 0.85, with subscale reliabilities ranging from 0.74 to 0.81, supporting its internal consistency in this sample.

### 2.4 Data collection

The survey was conducted using the internet data collection tool Questionnaire Star (www.wjx.cn). The corresponding author distributed the survey link to hospital leaders who then forwarded it to the nurses. To prevent spam submission, only participants with the same IP addresses or WeChat IDs were able to submit an online survey once. All data, including WeChat IDs and IP addresses, were used solely for research purposes and accessible only to the researchers. The expected completion time for all survey items was 10 min based on the total number of items. To ensure data accuracy, we excluded questionnaires completed in less than 3 min or more than 20 min, as well as those exhibiting random responding, failing consistency checks (e.g., contradictory responses to regular and reverse-scored items), or failing embedded attention-check items that instructed participants to select a specific response.

### 2.5 Data analysis

The data were entered into SPSS 18.0 (IBM Corp., Armonk, NY, USA) for analysis. Constant variables are expressed as means and standard deviations. Categorical variables are presented as figures and percentages. Univariate analyses were conducted to examine the relationship between participants' demographics and meaning in life. Pearson's correlation coefficients were calculated to explore the relationships between meaning in life, death anxiety, and attitudes toward caring for the dying. To compare the scores between different groups, *t*-tests and one-way analysis of variance (ANOVA) were used. In the multivariate regression analysis, variables that showed a significance level of *P* < 0.05 in the univariate analysis were selected as potential independent variables. Non-continuous predictor variables were coded using dummy coding before inclusion in the regression analysis.

## 3 Results

### 3.1 Characteristics of participants

In total, 1,698 questionnaires were completed, and 1,388 of them were deemed valid, resulting in an effective return rate of 81.7%. The participants were predominantly females (98.1%). The age group of 31–40 years comprised nearly 52.2% of the participants, and of these, 74.0% were married. Nearly half had junior titles (51.2%) and worked in tertiary-grade hospitals (56.6%). Most participants were not specialized nurses (69.0%) or had not received death education training (69.5%). Detailed demographic data are presented in Table 1.

**Table 1:** Characteristics of participants.

**Variable**	**N**	**%**	***t/F***	***P***
Gender			2.804	0.005
Female	1,361	98.1		
Male	27	1.9		
Age			10.674	<0.001
<30 years	443	31.9		
31–40 years	724	52.2		
41–50 years	178	12.8		
>50 years	43	3.1		
Ethnic group			1.287	0.198
Han ethnic group	1,344	96.8		
Others	44	3.2		
Marital status			8.032	<0.001
Unmarried	308	22.2		
Married	1,027	74.0		
Divorced or widowed	53	3.8		
Educational level			0.740	0.477
Junior college	328	23.6		
Bachelor degree	1,043	75.1		
Master degree and above	17	1.2		
Title			27.691	<0.001
Junior title	711	51.2		
Intermediate title	571	41.1		
Senior title	106	7.6		
Departments			0.285	0.837
Internal medicine	627	45.2		
Surgery department	269	19.4		
Acute and critical care unit	83	6.0		
Others	409	29.5		
Grade of hospital			3.198	0.023
Tertiary Grade A (Class III-A)	786	56.6		
Tertiary Grade B (Class III-B)	344	24.8		
Secondary Grade A (Class II-A)	165	11.9		
Secondary Grade B and below (Class II-B or Class I hospitals)	93	6.7		
Responsible institution			0.224	0.880
Nation	125	9.0		
Province	164	11.8		
City	659	47.5		
Others	440	31.7		
Nature of the hospital			0.577	0.564
General hospital	1,032	74.4		
Specialized hospital	356	25.6		
The location of hospital			0.548	0.701
Western Sichuan	91	6.6		
Chengdu	196	14.1		
Eastern Sichuan	79	5.7		
South Sichuan	791	57.0		
Northern Sichuan	231	16.6		
Position			13.955	<0.001
Responsible nurse	1,071	77.2		
Nursing team leader	145	10.4		
Head nurse	160	11.5		
Deputy director/director of nursing	12	0.9		
Average monthly income			8.548	<0.001
<3,000 RMB	100	7.2		
3,000–6,000 RMB	796	57.3		
6,001–10,000 RMB	451	32.5		
>10,000 RMB	41	3.0		
Whether a specialized nurse?			2.273	0.023
Yes	430	31.0		
No	958	69.0		
Religious belief			2.177	0.030
Yes	69	5.0		
No	1,319	95.0		
Have been bereaved within one year?			1.851	0.064
Yes	223	16.1		
No	1,165	83.9		
Average number of terminally ill patients cared for per month			0.087	0.917
0	782	56.3		
1–5	536	38.6		
>5	70	5		
Whether have received death education training			3.496	<0.001
Yes	424	30.5		
No	964	69.5		

### 3.2 Bivariate analyses of demographic variables and meaning in life

Bivariate analysis results demonstrated that gender, age, marital status, title, grade of hospital, position, average monthly income, whether a specialized nurse, religious beliefs, or having received death education training were significantly associated with a sense of meaning in life (*P* < 0.05) (Table 1).

### 3.3 Relationship between meaning in life, attitude toward care for dying, and death anxiety

The relationship between meaning in life, attitude toward care for dying, and death anxiety are presented in Tables 2, 3. The results of the study showed that the MLQ had a total score of 50.37 ± 9.30, FATCOD-B had a total score of 100.35 ± 10.88, and T-DAS had a total score of 47.40 ± 8.20. We first performed a correlation analysis to explore the relationship between meaning in life, attitudes toward caring for the dying, and death anxiety. According to the results, meaning in life had a negative relationship with death anxiety (*r* = −0.074, *P* < 0.01) and meaning in life a positive relationship with attitude toward care for dying (*r* = 0.343, *P* < 0.001).

**Table 2:** The score of scales.

**Variables**	**Minimum**	**Maximum**	**Mean ±SD**
Total score of MLQ	14.00	70.00	50.37 ± 9.30
The presence of meaning	5.00	35.00	24.90 ± 5.14
The search for meaning	6.00	35.00	25.46 ± 4.92
Total score of FATCOD-B	72.00	140.00	100.35 ± 10.88
Attitude toward the interests of dying person	12.00	30.00	22.96 ± 2.88
Attitude toward the caring for dying person	6.00	30.00	20.77 ± 3.87
Attitude toward the necessity of family support	10.00	22.00	17.13 ± 1.50
Attitude toward fear of caring of dying person	6.00	25.00	14.51 ± 2.90
Attitude toward communication with the dying person	7.00	20.00	15.51 ± 2.13
Attitude toward caring for the dying person's family	3.00	15.00	9.47 ± 2.16
Total score of T-DAS	19.00	74.00	47.40 ± 8.20
Emotion	6.00	30.00	16.96 ± 3.91
Stress with pain	4.00	20.00	15.17 ± 3.07
Time awareness	2.00	10.00	6.20 ± 2.07
Cognition	3.00	15.00	9.06 ± 2.59

**Table 3:** Relationship between meaning in life, attitude toward care for dying, and death anxiety.

**Outcome variables**	**Mean**	**SD**	**1**	**2**	**3**
1.meaning in life	50.37	9.30	1.00		
2.Death anxiety	47.40	8.20	−0.074^a^	1.00	
3.attitude toward death	100.35	10.88	−0.343^a^	0.188^a^	1.00

^a^*P* < 0.01.

### 3.4 Multiple linear regression analysis

Statistically significant demographic variables, including gender, age, marital status, title, hospital grade, position, average monthly income, specialized nurse status, religious beliefs, death education training, death anxiety, and attitudes toward care for dying, were identified through a *t*-test, ANOVA, and Pearson correlation analysis. These variables were then included as independent variables in a multivariate linear regression analysis, with a sense of meaning in life as the dependent variable. The results of the multiple linear regression are presented in Table 4. The regression model yielded significant findings, with (*F* = 25.364, *P* < 0.001), indicating a good fit between the linear regression equations and the data. The findings indicated that attitudes toward caring for the dying and death anxiety were significant influencing factors on nurses' sense of meaning in life (*P* < 0.001). Participants with religious beliefs had a significantly higher level of meaning in life compared to those without religious beliefs (β = −0.060, *P* < 0.05). Furthermore, being married was found to have a positive impact on the sense of meaning in life compared to being unmarried (β = 0.069, *P* < 0.01). A senior title had a significantly greater influence for participants on the sense of meaning in life compared to those with a junior title (β = 0.112, *P* < 0.001). Additionally, participants working in a Secondary Grade B and below hospital had a lower level of meaning in life compared to those in a tertiary grade A hospital (β = −0.067, *P* < 0.01). Moreover, being a head nurse had a positive impact on the sense of meaning in life, compared to other nurse grades (β = 0.058, *P* < 0.05).

**Table 4:** Multiple linear regression with predictor variables.

**Variables**	**B**	**SE**	**β**	**t**	***P***	**95% CI**
Constant	30.461	3.694	-	8.246	<0.001	
FATCOD-B	0.269	0.022	0.315	12.409	<0.001^*^	(0.227, 0.312)
Death anxiety	−0.060	0.026	−0.118	−2.259	0.024	(−1.218, 1.335)
Religious belief	−2.563	1.063	−0.060	−2.410	0.016	(−4.648, −0.477)
married	1.466	0.549	0.069	2.671	0.008	(0.389, 2.542)
Senior title	3.930	1.016	0.112	3.869	<0.001^*^	(1.937, 5.923)
Secondary grade B and below	−2.506	0.938	−0.067	−2.671	0.008	(−4.346, −0.665)
Head nurse	1.682	0.826	0.058	2.036	0.042	(0.061, 3.302)

^*^p < 0.001.

*F* = 25.364, *p* < 0.001, *R*^2^ = 0.260 (original), *R*^2^ = 0.249 (adjusted).

B, unstandardized regression coefficient; SE, standard error; CI, confidence interval; β, standardized regression coefficient; VIF, variance inflation factor.

## 4 Discussion

This is the first large-sample study to explore the current situation of meaning in life and its influencing factors among Chinese nurses. The results emphasize the significant correlation between attitudes toward care for the dying, death anxiety, religious beliefs, job title, marital status, grade of hospital, and position, which influence meaning in life. Overall, the findings provide new insights into the psychological responses of nurses working in the Chinese cultural environment, where discussions about death remain relatively limited.

The average MLQ score in this study indicated a moderate level of meaning in life among nurses. Compared with findings in other healthcare populations, the level observed here was slightly lower, which may reflect the high workload, stress, and emotional burden inherent in clinical nursing roles (Chuanyu, 2022; Shuhan et al., 2022; Xiuzhi, 2020; Yexiang et al., 2023). Meaning in life is closely related to nurses' mental health and emotion regulation (Chen et al., 2022). It plays a crucial role in enabling nurses to cope with adverse psychological issues, deliver humanistic care at work, and provide person-centered care (Zifen, 2015), therefore, it is essential to closely monitor meaning in life.

Consistent with theoretical expectations, meaning in life was positively correlated with attitudes toward caring for the dying. This suggests that nurses who perceive greater meaning in their work and personal life may experience a more grounded perspective on death and be more emotionally prepared to support dying patients (Maffly-Kipp et al., 2024). The positive association between MLQ and FATCOD-B may be interpreted through the lens of existential psychology, which suggests that individuals with a strong sense of purpose are more likely to view caring for the dying as meaningful rather than distressing (Dursun et al., 2022). Previous international studies have reported similar trends, supporting the idea that meaning in life may serve as a buffer that enhances motivation and compassion in end-of-life care (Bernard et al., 2017). Furthermore, in the Chinese cultural context—where death remains a sensitive and often avoided topic—having a strong sense of meaning may be especially important in enabling nurses to approach dying patients with acceptance and confidence (Zhang et al., 2025). This highlights the potential value of meaning-centered training or reflective interventions to strengthen nurses' attitudes toward caring for the dying.

This finding demonstrated that higher levels of death anxiety are associated with lower perceptions of life meaning. Nurses are frequently exposed to patient suffering, terminal illness, and death—circumstances that may intensify existential reflection and trigger emotional distress (Nia et al., 2016). In Chinese culture, where death remains a taboo subject, such exposure can exacerbate internal conflict and anxiety when coping with death (Zhang et al., 2024). Death anxiety may disrupt cognitive processing and lead to avoidance behaviors, thereby weakening nurses' ability to derive purpose and value from their work (Khajoei et al., 2022). Conversely, those with lower death anxiety may have developed more mature death attitudes, which allow them to engage with end-of-life care more meaningfully and with less psychological burden. Unlike Western societies where death education has been integrated into nursing curricula for decades, death-related training in China remains limited and inconsistent (Ledesma et al., 2023). These results suggest the need for targeted interventions—such as death education (Kim et al., 2024), reflective practices, and psychological support—to help nurses confront and manage death anxiety, thereby fostering a stronger and more stable sense of meaning in life (Glaw et al., 2017).

Little prior research has explored the relationship between religious beliefs and nurses' meaning in life (Womick et al., 2022). The results indicated that nurses with religious beliefs exhibited higher levels of meaning in life compared than those without religious beliefs. In China, Buddhism is the dominant religion, and there is a common Buddhist saying that “saving a life is better than building a seven-floor pagoda.” Consequently, nurses with religious beliefs may feel a greater sense of value in life and stronger professional identity while caring for their patients (Xu, 2021).

The current study validated the influential role of marital status in the sense of meaning in life, which is consistent with previous research. Previous studies have demonstrated that married nurses exhibit higher levels of overall meaning in life and a sense of existential significance compared to unmarried nurses (Chuanyu, 2022; Nowicki et al., 2020). A close and stable marital relationship can enhance an individual's sense of security and contribute to a deeper understanding of the meaning in life. Additionally, the couple can further enrich their individual senses of meaning in life through reciprocal encouragement and support.

The study findings revealed that nurses with senior titles demonstrated a higher level of meaningfulness in life than those with junior titles. Additionally, nurse managers exhibited a higher level of meaningfulness in life than responsible nurses. These findings align with those of some studies (Xiuzhi, 2020) but contradict others (Shuhan et al., 2022; Yexiang et al., 2023). Several factors may help explain these discrepancies. Senior nurses and nurse managers often possess greater professional autonomy, stronger clinical competence, and richer experience, which can enhance their sense of mastery and professional identity—key contributors to meaning in life (Pursio et al., 2021). By contrast, studies with opposite findings may include institutions where administrative burden is heavier or career advancement is limited, reducing the fulfillment typically associated with seniority. Additionally, variations in organizational culture and role expectations across regions or hospital systems may influence how professional titles shape nurses' sense of purpose. Based on these insights, hospitals may consider practical measures such as strengthening mentorship and skill-development opportunities for junior nurses, clarifying promotion pathways, and optimizing job design to balance administrative work with meaningful clinical practice (Kohnen et al., 2024). Supportive organizational strategies—such as peer support, reflective practice activities, and recognition of clinical contributions—may further enhance meaning in life across different professional levels (Oshodi et al., 2019).

The grade of the hospital in which the nurse worked also had a significant impact on the nurse's level of meaning in life, with nurses working in Secondary Grade B hospitals and below having a lower level of meaning in life than those working in Tertiary Grade A hospitals. This could be attributed to the fact that nurses in Tertiary Grade A hospitals are responsible for treating patients with complex and critical conditions, thereby encountering greater challenges in their work (Sibuea et al., 2024). However, when nurses possess the skills to effectively manage the complexity of their conditions and successfully save patients' lives, they experience a greater sense of fulfillment and find more meaning in their own lives.

### 4.1 Novelty and contribution

This study makes several novel contributions to the field. First, it employed a multi-center design, including 60 hospitals across Sichuan Province, which enhances the representativeness of the sample and provides insights across diverse clinical settings. Second, it specifically focuses on practicing nurses rather than nursing students or patients, capturing the psychological experiences of a professional group frequently exposed to high workloads, complex interpersonal dynamics, and repeated encounters with death. Third, this study considers the Chinese cultural context, where death is traditionally viewed as taboo, offering a culturally sensitive perspective on how death attitudes and anxiety influence meaning in life. Finally, the study simultaneously examines the interplay of sociodemographic factors, death anxiety, and attitudes toward caring for the dying, providing a more comprehensive understanding of the determinants of life meaning among nurses. Highlighting these aspects emphasizes the study's unique contribution to both nursing psychology and occupational health research.

### 4.2 Practical implications

These findings have practical implications for nursing management and policy. Interventions such as death education programs, psychological support and counseling, professional development opportunities, and organizational strategies to improve workplace support could enhance nurses' sense of life meaning. Culturally tailored programs that address death attitudes and foster resilience may further support nurses' wellbeing and professional fulfillment, ultimately benefiting patient care.

### 4.3 Limitations

This study has some limitations. First, the use of convenience sampling resulted in uneven population distribution across different regions, potentially influencing the reliability of the results. Second, the inherent limitations of cross-sectional studies may hinder the ability to draw causal inferences from the results. Third, the study's focus on nurses in Sichuan Province limited the geographical representation of the study population, suggesting the need for a nationwide study in the future. Fourth, the regression model explained only 24.9% of the variance in nurses' meaning in life. Although this level of explanatory power is typical for studies examining complex psychosocial constructs, it indicates that additional factors may influence life meaning. Unmeasured variables—such as work-related stress, interpersonal support, coping strategies, personality traits, and organizational environment—could further contribute to variations in life meaning.

## 5 Conclusion

The findings of this study indicate that nurses in Sichuan Province have a moderate level of meaning in life. Various factors, such as attitudes toward caring for dying patients, marital status, religious beliefs, title, hospital level, and position, were found to influence nurses' meaning in life. Administrators and regulatory agencies can use these personality traits to develop targeted intervention programs that enhance the meaning in life and help nurses discover the value of their lives. This could motivate nurses to provide better care and services to their patients.

## Data Availability

The original contributions presented in the study are included in the article/supplementary material, further inquiries can be directed to the corresponding authors.
